# Market-orienting reforms in rural health care in Sweden: how can equity in access be preserved?

**DOI:** 10.1186/s12939-018-0819-8

**Published:** 2018-08-17

**Authors:** Linn Kullberg, Paula Blomqvist, Ulrika Winblad

**Affiliations:** 1 Department of Public Health and Caring Sciences, Uppsala University, Uppsala, Sweden; 2 Department of Government, Uppsala University, Uppsala, Sweden

**Keywords:** Rural health care, Marketization, Patient choice, Health care planning, Health governance, Sweden

## Abstract

**Background:**

Health care provision in rural and urban areas faces different challenges. In Sweden, health care provision has been predominantly public and equitable access to care has been pursued mainly through public planning and coordination. This is to ensure that health needs are met in the same manner in all parts of the country, including rural or less affluent areas. However, a marketization of the health care system has taken place during recent decades and the publicly planned system has been partially replaced by a new market logic, where private providers guided by financial concerns can decide independently where to establish their practices. In this paper, we explore the effects of marketization policies on rural health care provision by asking how policy makers in rural counties have managed to combine two seemingly contradictory health policy goals: to create conditions for market competition among health care providers and to ensure equal access to health care for all patients, including those living in rural and remote areas.

**Methods:**

A qualitative case study within three counties in the northern part of Sweden, characterized by vast rural areas, was carried out. Legal documents, the “accreditation documents” regulating the health care quasi-markets in the three counties were analyzed. In addition, interviews with policy makers in the three county councils, representing the political majority, the opposition, and the political administration were conducted in April and May 2013.

**Results:**

The findings demonstrate the difficulties involved in introducing market dynamics in health care provision in rural areas, as these reforms not only undermined existing resource allocation systems based on health needs but also undercut attempts by local policy makers to arrange for care provision in remote locations through planning and coordination.

**Conclusion:**

Provision of health care in rural areas is not well suited for market reforms introducing competition, as this may undermine the goal of equity in access to health care, even in a publicly financed health care system.

## Background

A significant part of the world’s population lives in rural areas. There are vast and remote rural areas in Australia, Canada, and the United States, and considerable areas with rural characteristics can be found in European countries such as the United Kingdom, Sweden, Norway, and Finland. Providing health services to people residing in rural areas is associated with several challenges, such as long traveling distances, difficulties in recruiting staff, and low availability of specialized care. The literature on rural health care points to coordination, cooperation between health units, and adaption to local needs as key strategies to overcome these challenges. Typically, such measures require public investment and planning [1–4]. Thus, health services provision to populations in rural areas can be seen as a case of *market failure*; investment costs are too high and expected profits too low to attract private capital [5, 6]. In recent decades, however, many Western health care systems have undergone processes of marketization. This implies that reforms have been introduced which have strengthened financial incentives through performance–related payment systems, strengthened elements of competition, widened opportunities for patient choice, and, in some cases, increased the share of private providers [7–9]. Objectives behind marketization reforms in health care have typically included the increase of economic efficiency and cost control, quality development through entrepreneurialism, and the strengthening of the role of patients [10–12].

In rural areas, it is hard to find suitable conditions for market competition given the low number of providers and the long geographical distances between them [13–15]. In addition, the tendency of private care providers to establish in urban, densely populated areas presents a risk to rural care provision as this tends to reinforce the propensity of funds being allocated foremost to the cities, leaving patients in the countryside more deprived. This logic is apparent in systems where “money follows the patient,” which makes it harder for health authorities to financially compensate providers located in areas with fewer inhabitants [16–18]. Systems where public and private health providers coexist and compete for patients may also present difficulties for health provision in rural areas, as this reduces the ability of public authorities to coordinate care activities and promote cooperation between different care providers. For these reasons, marketization of health care in the form of privatization of provision or patient choice has sometimes been perceived as a threat to principles of equity in access and needs-based distribution of care [19, 20], which are central in many European health care systems.

One country where the inherent tension between rural health care provision and marketization reforms has become apparent is Sweden. As a universal, public health care system of National Health System type, the goal of equity in access to health services plays a central role in Swedish health care. At the same time, the system has recently undergone a process of marketization, particularly in primary care. In 2010, the Primary Care Choice Reform (*vårdvalsreformen*) was introduced that replaced the previous public population-based organization of primary care services with a system based on patients’ free choice of primary care provider (public or private) in the country. Free establishment of private care providers was introduced, in effect depriving the local health authorities of control over the geographic location of primary care centers. The Primary Care Choice Reform thus replaced the previous system of government distribution of resources with a market-driven system where the choices of patients and locations of providers determine the allocation of resources [21]. As Sweden is a country with vast rural areas, particularly in the north, critics of the reform feared it would undermine the ability of local health authorities to uphold access to primary care services for populations in such areas, thereby undermining the principle of equity in access in Swedish health policy.

In this article, we investigate whether such fears have proved warranted by examining the effects of the Primary Care Choice Reform when it comes to private establishment and implications for resource distribution in rural areas. The main research question asked in the article is how health authorities in rurally located counties have responded to the Primary Care Choice Reform and which, if any, measures have been taken by them in order to uphold the goal of equity in access to care within their areas. In order to answer the question, three case studies of the implementation of the Primary Care Choice Reform in rural counties were conducted. An important condition of the case study setting is that local health authorities in Sweden, the county councils, enjoy a substantive degree of autonomy in organizing the provision of services as long as national legislation is honored, a fact that provided rural counties with a certain flexibility in implementing the Primary Care Choice Reform. The case studies were guided by two more precise questions: first, which, if any, challenges were perceived by local health authorities to be associated with implementing the reform; and, second, what specific measures, if any, were taken to preserve access to care for patients in remote locations while at the same time opening up the local system of health provision to private competition?

The findings presented in the article indicate that rurally located counties in Sweden did use their autonomy to modify and adapt the market-orienting Primary Care Choice Reform in order to protect access to health care in the rural areas within their jurisdictions. The most common measures were to design local accreditation rules so as to ensure that all primary care providers offered a broad scope of care services, to choose a high share of capitation-based financial reimbursement (rather than reimbursement based on patient visits), and create a special ‘rural’ allowance for providers with patients living in remote areas.

### Health care provision in rural areas

There is no generally accepted definition of ‘rural’ and several definitions and categorizations are used in parallel. Most definitions include both population- and geography-based factors [22]. In England and Wales, an area is defined as rural if it falls outside of settlements with more than 10,000 residents. In Sweden, the Swedish National Rural Development Agency defines rural as “areas with more than 45 minutes travel to the closest urban center with more than 3,000 inhabitants.” [23]. The variety of rural definitions reflects the scope and complexity of the concept and thus the need to consider contextual aspects when defining, studying, and developing rural health [24, 25].

It has previously been noted that rural and urban areas present different challenges for an optimal design of health care services [2]. Among the most frequently discussed problems regarding health care provision in rural regions are the tendency for lower health status in rural populations compared to more urban settings. Examples of challenges for rural health care provision are: population ageing; lack of resources; poor access to health care services due to long travel distances; lack of public transportation; and shortage of health care professionals, particularly doctors [25–28].

Since the 1990s, research on rural health care provision has focused on possible ways to overcome the hindrances to provision of health services in remote areas. Generally, the literature suggests that flexibility and ability to adapt the organization of service provision to the local context are key to overcoming some of the challenges to rural health care provision. Asthana and Halliday [2] conclude that optimal approaches to service organization and delivery in rural areas are likely to vary from area to area. Wakerman et al. [1] conclude that ‘one size doesn’t fit all’ when it comes to models of primary care provision in rural settings.

A recurring theme in the literature on rural health care provision is the importance of *coordination and cooperation* between different public actors involved in health promotion. The ‘whole-system-approach’ accentuates the importance of coordination and cooperation between community health care services, social care, specialized care, and acute care hospitals. Although coordination is important beyond rural settings, the literature suggests that the potential cost-savings that can be made by improving coordination and avoiding service duplication might be of particular importance in rural areas [21]. A second way to overcome the challenges associated with rural health care provision noted in the literature is *flexibility in the use of staff*. A common example is to expand the role of nurses, which has been found to be the case in several studies, where nurses performed tasks normally carried out by other professions [2, 27]. Inter-professional cooperation is also mentioned by some as an important strategy for rural health care provision [25]. A third solution to rural challenges such as long distances and lack of public transportation has been *professional outreach*, implying that health professionals travel out in mobile teams (such as by car, boat, or helicopter) to remote populations [2].

Finally, the usage of *telemedicine and e-Health*, in the form of online diagnosis or medical consulting, has been proposed as an important means to address the health care needs of patients in remote locations [2, 27]. Telehealth reduces the need for patients to travel as they can be treated in their local community. For instance, the use of fetal telemedicine for anomaly scanning reduces the need for pregnant women to travel long distances [2, 29]. E-Health has also been found to be an effective tool to expand knowledge and educational opportunities among rural health professionals [2, 27]. In sum, it can be noted that strategies suggested to improve rural health care provision include local adaptions of service provision systems, innovative cooperation between different actors, flexible use of medical staff and the development of telehealth.

Implicit (sometimes explicit) in this discussion is the assumption that public planning and coordination are necessary means to reach a sufficient level of care provision in rural areas. Conditions for market-based health care provision, building on economic incentives and competition among care providers, are typically described as poor in rural areas. Competitive mechanisms are hard to sustain, given the low number of providers and the long traveling distances for patients. Economies of scale, generating efficiency, are hard to achieve [15, 30]. Some studies have concluded that private companies tend to disfavor rural areas, as such markets are not seen to be as attractive as urban ones due to lower population density and more sickly patients [14]. Other studies have noted that the tendency of private care providers, including self-employed General Practitioners, to locate mainly in more densely populated areas may lead to public resources being channeled away from rural regions into the cities, thus reinforcing differences in access to care between cities and the countryside [31–33]. Hence, it may be concluded that market-based provision of care, especially if this entails private care providers or providers who are free to decide their location and receive funding based on the number of patients they manage to attract, can pose a threat to the goal of equal access to care for populations in rural areas. Equal access to health care is a common goal in health policy and can be understood to mean that there should be equal opportunities for individuals to obtain health care services when in equal need for care, regardless of social class, geographical residence, ethnicity or gender [34, 35]. In the case of geographical residence, it is natural that there is some variation in access between areas. However, the goal should be, according to Olivier and Mossialos, for health authorities to ensure supply of a specified level of health services to which all individuals within an area should have access without too much cost or personal inconvenience [35]. So far, however, there are few studies which have investigated systematically the impact of marketization reforms on the supply of health care services in rural areas and the implications for geographical equity.

### Case study context: The primary care choice reform in Sweden

The Swedish health care system is publicly funded through income taxation and provides automatic coverage against virtually all health risks to the whole population. The principle of equity is prominent in the legislation and health services are distributed solely on the basis of medical need. Private health insurance is rare. The system is heavily decentralized, as 21 local governments, county councils, are responsible for funding and providing health care services. Most services are provided through public facilities, owned and operated by the counties themselves. Local income tax, levied by the counties, makes up about 80% of the system’s funding, the rest comes from grants from the national government (17%) and, to a smaller extent, user fees (3%). The county councils enjoy a long tradition of local autonomy in organizing the provision of services, which also includes regulation of private health care providers and the setting of patient fees. The highest governing body in the county councils is the county parliament, consisting of democratically elected representatives of local parties. The parliament appoints the county council board, representing the political majority in elections preceding such appointments.

The primary care sector in Sweden was until the 1990s organized as a virtual public monopoly. Following legislation in 1970, primary care provision has been organized through primary care centers, which are community-based and cater to the basic medical needs of the population within a defined geographical area. Primary care centers have typically been responsible for basic medical treatment, nursing care, preventive care, and rehabilitation. Some also provide prenatal- and maternity care. Typically, a primary care center consists of a multidisciplinary team of General Practitioners (GPs), nurses, midwives, physical therapists and psychologists. Its average size in 2012 was 4–6 GPs, although the number varies substantially, even within counties [8]. Prior to 2010, the funding of primary care centers through the counties was usually organized on the basis of yearly budgets and the staff was salaried. The location of primary care centers was subject to careful planning by the counties and establishment decisions were based on estimations of health needs [36].

The Primary Care Choice Reform (*Lag om valfrihetssystem)* [37] introduced in 2010 by a center-right government drastically changed the governing logic of Swedish primary care. The aim of the reform was to support innovation, private entrepreneurship, and quality development in the primary care sector through the introduction of market mechanisms such as competition and consumer choice. The choice between care providers was also believed to empower the patients [38]. The reform, however, did not alter the underlying principles of public funding and universal access to health care in Sweden but began a partial privatization of provision in that private actors were given the right to freely establish in the system and compete with public primary care centers for funding. Fair market competition was a central principle in the legislation, which implied that the county councils had to allocate resources among the competing providers solely based on pre-set funding formulas that were the same for all providers. The reform thus led to a change from previous budget systems to a new funding system based on a mixture of per-capita payments and, in some counties, combined with a fee-for-service part. In most counties, per capita payments became risk-adjusted, taking into consideration factors like age, sex, and socio-economic characteristics. This funding system implies that public and private producers compete on equal terms and no provider is guaranteed any resources [21, 32, 39]. The regulatory part of the reform is based on a system of accreditation, where the county councils formulate requirements for establishment of providers in their county as well as the terms for funding and patient listing. It is in the accreditation documents that the demands for quality are regulated, such as regarding staff competence, access, and quality improvement work. The conditions for accreditation are formulated as a call for tender, which, after a contract is entered between the county councils and the accredited care providers (public as well as private), comes to constitute the regulatory framework governing the local choice system.

When the first proposal for the Primary Care Choice Reform was presented in April 2008, it stated that conditions for accreditation of providers should be set by the central government and hence be the same for all county councils. However, this proposal was met with such strong protests, foremost from the county councils themselves but also from other consultative expert bodies, that it was eventually withdrawn. In the revised proposal, presented six months later, the counties were given freedom to formulate local accreditation systems themselves, albeit while honoring the principles of free and fair competition [21]. This allowed them to adapt regulatory frameworks, such as regarding funding formulas and the kinds of services that providers were required to offer, to varying local conditions. This also implies that the county councils can designate what services local primary care providers are obliged to provide, and whether the scope of services is limited or broad, including, for example, maternity health and psychotherapy in addition to basic GP and nurse services. Another innovation was that counties with rural areas were allowed to offer additional funding to providers establishing in such areas [38]. This adjustment meant that the county councils retained more of their traditional local autonomy, even if the reform was still generally unpopular with the counties, particularly those with left-wing political majorities. A commonly voiced concern was the implications for equity in access, as it was expected that most new private establishments would take place foremost in urban and more prosperous areas [38]. If so, this would run counter to the overriding goal in Swedish health care of equal access to health services for the whole population, as expressed in the statutory 1982 Health and Medical Services Act (*Hälso- och sjukvårdslagen*). In terms of geographic proximity, the legislative bill establishing the Act stated that health services should be “close and easily accessible” (*nära och lätt tillgänglig)* and that primary care centers in particular should be located so that they were easily accessible for all [40].

The effects of the Primary Care Choice Reform have been evaluated by several official agencies. Reports document a substantive increase in new private establishments, leading to an overall growth in the number of primary care centers [33, 41], about 29% in the years 2010–2017.^1^ In 2016, a little over 40% of all primary care centers were operated by private actors in Sweden, the majority of them for-profit firms.^2^ Some studies indicate that equity has been reduced by the Primary Care Choice Reform, as most establishments have occurred in densely populated areas [41], and in areas with a younger population [17]. However, no studies have explicitly focused on the effects of the reform in rural areas.

In sum, it is apparent that the Primary Care Choice Reform has altered the conditions for governance within the primary care sector. Most important, the autonomy of the county councils, which previously implied that they could decide independently whether and under what conditions to allow private providers, has been reduced. With the new legislation on choice systems, the county councils have also lost the ability to plan the geographical distribution of services and to provide extra resources to providers in remote areas. Important to note, however, is that the county councils remain legally responsible for upholding the principle of equal access for all citizens to care services as stated in the Health and Medical Services Act. This implies that the county councils are faced with two conflicting health policy goals: to ensure equal access to care services for all citizens within their districts, including those residing in remote areas, and to ensure the principles of free and fair competition for all care providers.

## Methods

The empirical study undertaken consists of three case studies investigating how local policy makers in county councils located in the northern part of Sweden implemented the 2010 Primary Care Choice Reform and how they addressed challenges regarding the preservation of access to care in remote areas. The case studies were conducted in 2013. Case studies are known to be an appropriate method when trying to uncover the reasoning behind political decision-making, in particular when it comes to how contextual factors such as the political environment, or structural conditions regarding geography, population, or economy, impact policy choices [42].

### Case selection

In the first part of the case selection process, all Swedish county councils with large rural and remote areas were identified. Given the Swedish definition of rural areas, no county can be defined as only rural or urban. However, in line with the Swedish Agency for Growth Policy Analysis' definition of rurality, six of Sweden’s 21 counties and regions are characterized by large rural areas (Norrbotten, Västerbotten, Västernorrland, Jämtland, Dalarna, and Värmland) [43]. These are all located in the northern part of Sweden.

**Fig. 1:**
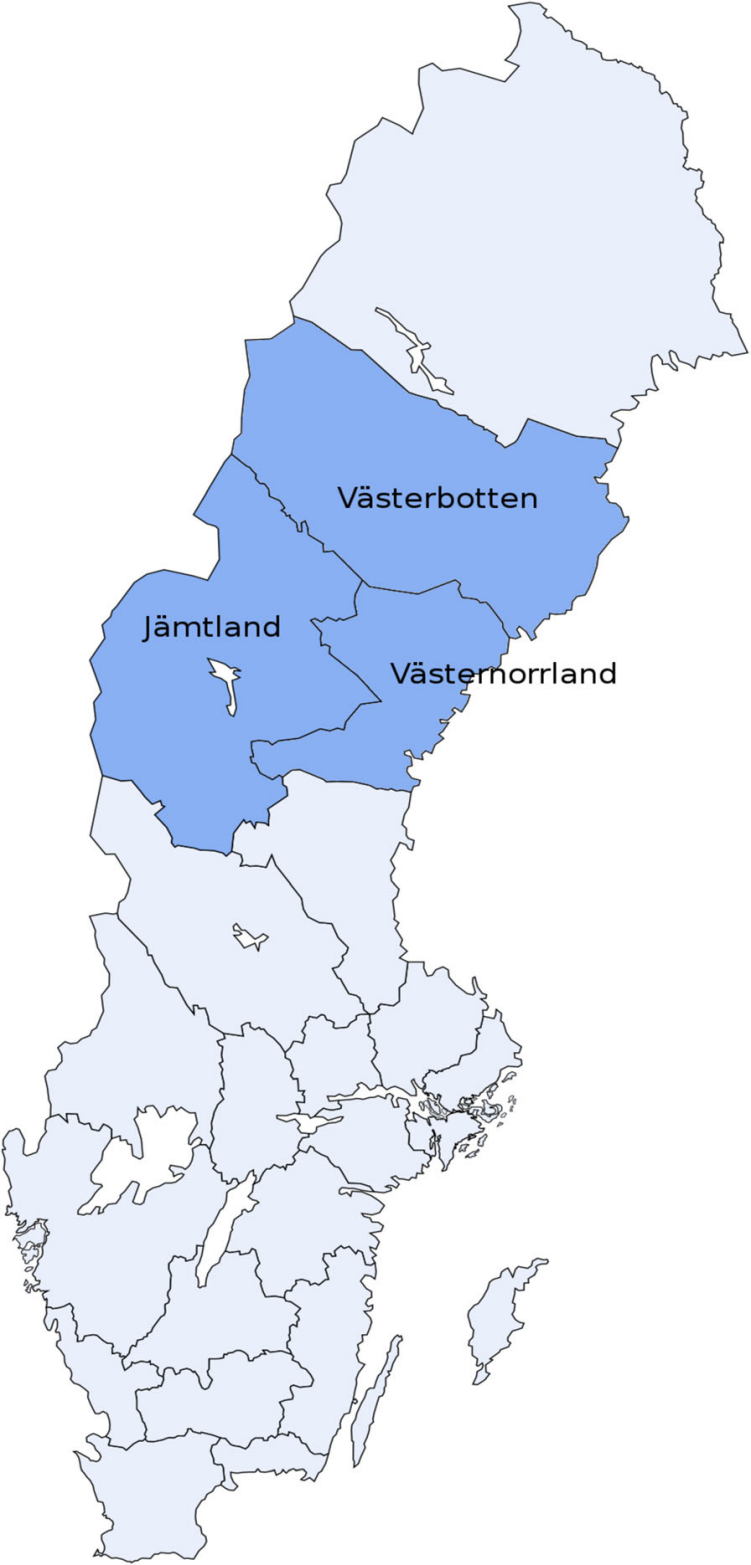
The three selected county councils

In the next step, a selection of three county councils out of the six was made (see Fig. 1). In order to get a broad and varying sample in accordance with a maximum variation strategy (see [44, 45]), cases were selected which varied on the following characteristics: share of private providers; political governing majority in the county councils; and, mix of rural and urban areas. The three chosen cases were Jämtland, Västerbotten, and Västernorrland (see map, Fig. 1). *Jämtland,* located in the northwestern part of Sweden, is heavily forested and is the county in Sweden with the highest number of primary care providers located in rural areas. *Västerbotten,* the second largest county in Sweden by surface area, stretches from the eastern coast to the western border with Norway and is characterized by a mixture of relatively densely populated urban areas along the coast and vast rural areas inland. *Västernorrland* is smaller and distinct from other rural counties in that it was governed by a central-right political majority in the county council during the time of the study. In 2013, it was the rural county with the largest share of private primary care providers. These cases, while all being rural, offer some variation with regard to the geographical distribution of the population, the share of private providers, and the political orientation of local policy-makers.

### Research methods and materials

This qualitative study was based on key informant interviews and document analysis.

The main method used for analyzing the data was directed content analysis. This implies a deductive analytical approach, where previous studies guide the formulation of research questions, and analysis of empirical materials draws on pre-established concepts known to be relevant to the issue at hand [46]. For the purposes of this study, an analytical framework was constructed which enabled an analysis of different ways in which county council policy makers could act to preserve equity in access to care for residents in remote rural areas after the implementation of the Primary Care Choice Reform. Based on previous research [18, 47] as well as domestic policy discussions [48], the framework identifies three possible measures which the county councils could use to this end: 1) The scope of services all primary care providers in the county were asked to provide; 2) The regulation regarding how patients are listed at newly established primary care centers; and 3) The design of the reimbursement system. Based on these measures, our coding categories were; *scope and content*; *patient listing;* and *reimbursement.* We consider these three measures as means to operationalize the goal of geographical equity in access to care. Requirements by local authorities on extensive *scope and content* of services, including for instance rehabilitation services, home-based care or paramedical services, is thought to support geograpical equity in access as this model guarantees a broad range of services for people living far from the cities. A *patient listing system* that enables new providers to rapidly receive a lot of patients and thereby receive higher reimbursement is thought to favor urban establishment rather than rural. By designing the *reimbursement system* to provide extra payments for weaker socio-economic groups and extra allowances for establishments in more remote areas, the county councils could attract care providers to rural areas.

The empirical materials used in the study were of two main types: policy documents and interviews with local policy makers such as elected politicians and civil servants. The policy documents, referred to as ‘accreditation documents’ (*förfrågningsunderlag*), can be seen as constituting the main regulatory framework for the local implementation of the Primary Care Choice Reform in each county. These documents, between 75 and 100 pages long, reveal how local policy makers have chosen to formulate the conditions for the establishment of private care providers within their jurisdictions, for instance with regards to financial reimbursement and the types of services to be provided. By serving as a legally binding contract between the counties and the private providers, the accreditation documents constitute the main way in which the county councils can steer primary care after the 2010 reform.

The informants were identified through the county council web sites and through personal contact. Altogether nine interviewees were selected: two politicians and one civil servant in each county. The basis for the informant selection was the desire to get the views of the political majority as well as that of the main opposition party in each county. The highest ranking politician representing the political majority in a county council is mostly the chairperson of the health board. The highest ranking representative of the opposition in the county is usually the vice chairperson. Therefore, the chairperson and vice chairperson in each of the selected counties were selected. Given the relatively small size of the county councils, it was considered to be enough to interview two political representatives in each case. In addition to the political representatives, it was considered valuable to interview a civil servant responsible for the operational work of implementing the reform, as they are often more familiar with day-to-day management. In the end, however, a civil servant in one case declined to participate. The selected interviewees were contacted by e-mail with an attached information form containing information about the purpose of the study and a request to participate. The respondents were informed that their name would not be published, but due to their key positions it would be impossible to ensure total anonymity, a condition which all of the respondents accepted. All interviews were conducted by telephone between 22nd of April and 2nd of May 2013 and lasted between 40 and 70 min each. All interviews were, with the respondents’ permission, recorded and transcribed verbatim.

The interviews consisted of semi-structured questions regarding the interviewees’ perceptions and experiences of the implementation of the Primary Care Choice Reform. Three overriding questions guided the interviews: how the 2010 Primary Care Choice Reform was perceived by the informants; what, if any, specific local challenges they saw associated with implementing the reform in their own county; and, what measures or initiatives had been taken in order to protect equal access to care for all inhabitants in the county, including those living in remote areas. Semi-structured interviews were considered appropriate in order to give the respondents the opportunity to speak freely about the matter while still guiding the conversation so that the topic of the study would be covered. In the first step of the analysis, all transcripts were reviewed and text that appeared to match the guiding questions was highlighted. In the next step, all highlighted text was coded based on predetermined codes [46]. For the first two overriding interview questions, one code for each question was used. To code answers to the third question, the same analytical framework that had been used to code the contents of the accreditation documents was used, containing the three categories of *scope and content*; *patient listing* and *reimbursement*. Text related to the third question that could not be coded into any of the predetermined subcategories was coded into a new category named *other measures.* In the next step, the material under each category was summarized and relevant quotes were chosen to illustrate the results. The quotes were translated into English from Swedish by the authors. Changed or deleted text within the quotes was marked with brackets.

Once the analysis was completed, the research team met on several occasions to discuss and reconcile the interpretations of the findings. Some disagreement came up regarding the interpretation of how the counties used the listing system as a means to attract new providers. Consensus was achieved through discussing the definition of that particular code.

The second source of empirical information regarding the cases was interviews with key informants. The purpose was to gain a deeper understanding of how the potential conflict between the goals of the 2010 choice reform and the preservation of equity in access to care was perceived by the county councils and what types of measures had been taken to address it when the reform was implemented. The interviews were seen as complimenting the analysis of the formalistic documents [49].

## Results

### The case of Jämtland

The county of Jämtland is rather large at 50 km^2^, but has a small population of only 126,000 inhabitants; it has a population density of 3 people/km^2^. About half of the population lives in Östersund, which is a middle-sized city. The county has large remote and rural areas; some people would have to travel up to 250 km to visit their closest hospital. The left-wing Social Democratic party (*Socialdemokraterna*) has long been the largest political party, ruling together with the Left-wing (*Vänsterpartiet*) and the Green party (*Miljöpartiet*). The largest party in opposition is the Liberal-conservative party (*Moderaterna)*, which holds about 11% of the seats in the local parliament. When the Primary Care Choice Reform was introduced in 2010, Jämtland had 25 primary care centers, of which 23 were publicly operated and two private non-profits. After the new accreditation rules were introduced in 2010, one new private for-profit provider chose to locate in the county, in the small village of Hoting with 700 inhabitants.

The interviews conducted in Jämtland show that perceptions of the Primary Care Choice Reform are largely negative, particularly on the part of the representative for the governing Social Democratic party. She indicated that the political majority is not against the principle of free patient choice of providers but believes that the reform as a whole causes problem for the county since it means that the county loses its ability to steer and allocate resources in the most appropriate way:

“Before [the Primary Care Choice Reform] we could reallocate resources if we saw that a primary care center was in economic trouble. But if we are to reallocate to our own [public primary care centers] today, the same amount… must be given to the private providers as well.” (The political representative for the majority, Jämtland)

The civil servant interviewed agreed, stating that even if there is still some steering capacity, it is harder to implement changes while honoring the principles of fair competition and the accreditation rules: “it is difficult to make changes with short notice.” The political representative in opposition expressed the view that it is not the Primary Care Choice Reform per se that makes it hard to uphold equity in access to care in a county like Jämtland, but the geography itself, with the long distances for patients to travel:

“[Equity] is mostly a nice word on paper; it is impossible to achieve in a county like this. Then you need a hospital in every municipality: that is impossible!” (The political representative for the opposition, Jämtland)

It became clear from the interviews that the steady reduction in population size in the county makes it harder and harder to keep the primary care centers that now exist in the county, as the tax–base is gradually reduced. Adding further to the local economic hardships is the difficulty of recruiting permanent staff to the health centers. To solve the situation, the county has to rely on temporary medical personnel from commercial staffing agencies, which is significantly more costly than direct employment of ordinary staff.

#### Measures taken to preserve access to care in rural areas in Jämtland

The accreditation documents show that the county has chosen to demand a broad scope of services to be provided by all providers. The rules require all primary care centers to provide planned and unplanned medical services, maternal health services, child health services, rehabilitation, and psycho-social services such as counseling, some of which lie outside the basic package of services offered by most primary care centers in Sweden [50]. Interviewees in the county explain that a broad scope of services was considered necessary to promote accessible care in the remote areas:

“We wanted the new choice system to have the same broad scope of services in the rural areas as we had before the reform so that we still could have maternal health care services, child welfare centers, psychologists, etc., out in the countryside.” (Civil servant, Jämtland)

The politician representing the political majority gave the same type of explanation:

“We made this choice on behalf of our citizens, so that they would have access to a primary health care center near where they live.” (The political representative for the majority, Jämtland)

A second characteristic of the accreditation rules in Jämtland is that the funding formula for primary care providers has a high share of capitation (85%). This means that care providers are reimbursed predominantly for patient listings, and not for their actual visits. The capitation sum is risk-adjusted according to age and a care need index (CNI), commonly used in Sweden, that takes into consideration factors such as education, employment, and county of birth. Notably, the capitation formulas also contain a special ‘rural allowance’, based on the share of patients who have more than a certain distance to travel to the nearest hospital [50]. The interviews conducted in the county confirmed that rural allowances are considered the main reason why rural primary care centers are able to survive financially. The politician representing the opposition argued that the rural allowance made it more profitable to establish in rural areas, in effect disfavoring the providers in the cities:

“They [providers in rural areas] are better off, since they get more money, and that is why the private providers chose to establish in the rural parts…. …the Social Democrats said that this [the reform] is something for the bigger cities, but [here] it turned out to be the other way around.” (The political representative for the opposition, Jämtland)

When it comes to rules for patient listing, Jämtland has chosen a principle of ‘active re-listing’ instead of passive geographical listing, meaning that a newly established primary care center does not automatically receive patients from the local neighborhood; instead patients need to actively re-list themselves with the new primary care center in order for the provider to receive any capitation [50]. This makes it more difficult for new providers to get enough new patients quickly and can, therefore, be considered as something of a deterrent for a private establishment.

An additional measure used in the county of Jämtland to uphold primary care services in rural areas is cooperation with a municipality in one of the most remote parts of the county, where the 850 inhabitants have to travel 150 km to the closest primary care center. To provide them access to basic health care, the county council and local municipality co-finance a district nurse and an ambulance. This cooperation violates the principles of the Primary Care Choice Reform, in that the range of services offered is narrower than stipulated by the accreditation rules and that the funding is not based on the fixed capitation formula. The interviews reveal that the principle of fair competition is violated in Jämtland in other ways. It is disclosed that certain public providers are allowed to run at a loss, even though they, according to primary care choice legislation, should be closed down if they cannot enroll enough patients to cover their costs. Instead, the county allocates extra resources to them to cover their costs. In line with the principles of fair competition stated in the Primary Care Choice Reform, any additional resources allocated to one caregiver must also be given to all others, but according to the politician in opposition this does not always occur in practice, and, if it does, it is several years later, which in effect still offsets fair competition.

In sum, it appears that, in the case of Jämtland, the Primary Care Choice Reform was perceived by the political majority as a threat to the principle of equitable access to care for inhabitants in rural areas. As a result, several measures were taken by the county to prevent a resource drain from such areas to more urban ones and to compensate for the fact that it is typically more expensive to provide services in rural areas. Jämtland is also the only case where there had been no private establishments in the larger towns, only one in the countryside.

### The case of Västerbotten

Västerbotten is a county with an area of 55 km^2^ from the east coast to the Norwegian border in the west. The population is 260,000, twice the size of Jämtland’s. Most people (78%) live in the larger coastal cities. The largest city, Umeå, has grown significantly in the last decades, attracting inhabitants from the entire region. Hence, the county features a mixture of large rural areas and urban settings. The Social Democrats have a long history of dominance in the county and had over 40% of the votes in 2013, ruling together with the Left and Green parties. The right-wing Moderates have long been the biggest opposition party. When the Primary Care Choice Reform was implemented in 2010, Västerbotten county had 34 primary care centers, all public except two private for-profits. Following the reform, five new private health centers have established in the county, four in Umeå and one in another city, Lycksele.^3^

Participants from Västerbotten also expressed skepticism about the Primary Care Choice Reform. All interviewees, even the representative from the right-wing opposition, agreed that patient choice and competition are inappropriate in health care provision in rural areas since the patient base is too small. They considered it unlikely that a private provider would want to establish in a rural area. As explained by the civil servant:

“I have never heard of anyone [provider] willing to establish in, for example, Vilhelmina [a small town in the rural part of the county]. I would be super-surprised if someone showed interest, then I would wonder from where they get the money… …how they can run a business we now run with a deficit every week.” (Civil servant, Västerbotten)

The politician representing the political majority, the Social Democrats, argued that the reform has diminished the steering capacity of the county council as it is now bound by the accreditation rules. Even though it is possible to alter these rules, the representative pointed to the significant time lag between changes in, for example, the reimbursement formula and its effects. Another negative impact of the reform highlighted by him is that several innovations initiated by the county council in rural areas, like the development of e-health and new forms of cooperation with local municipalities, had to be terminated because of the reform since the new formula-based reimbursement system did not cover such activities.

A special challenge in Västerbotten county is the pronounced difference between the urban and rural areas. There are relatively large municipalities, like Umeå with 117,000 inhabitants, and very small ones, like rural Sorsele with only 3700 inhabitants. To find a regulatory framework, particularly regarding the scope of services, that is suitable in both settings is a challenge. Both the politician representing the left-wing majority and the civil servant stressed that there is a trade-off between offering a variety of choices for inhabitants in the bigger cities and preserving access to any kind of services in the rural areas.

The respondents in Västerbotten pointed out that the greatest challenge to health care provision in rural areas is the difficulty of recruiting medical staff, especially doctors, to rurally located health centers. Even though this was difficult prior to the primary choice reform, it is believed to have been made worse by the fact that the reform makes it harder to provide the resources needed to the rurally located health centers. As in Jämtland, the respondents talked about the vicious circle whereby primary care centers in rural areas are forced to rely on doctors from staffing agencies hired on a weekly basis, which results in much higher costs and undercuts high-quality care by making it harder to uphold continuity in the relationship between patients and GPs. The high cost of temporary staff, which can be three times the price of regular, salaried, staff, also means that such centers can afford fewer hours of scheduled doctors to divide between patients.

#### Measures to promote equity in access to care in Västerbotten

As in Jämtland, the accreditation rules in Västerbotten county demand a broad scope of health services to be offered by all providers. Primary care centers are required to provide planned and unplanned medical services, maternal and child services, rehabilitation services, preventive home visits for the elderly, and health examinations of asylum seekers [51]. All the interviewees emphasized the importance of meeting the goal of accessible health care for all of the population, regardless of where they live. One strategy used is to demand that primary care centers provide a broad scope of services so that people in rural areas can get easy access to all types of health care services. As in Jämtland, the listing rules state that if a new private provider is established, individuals living near that center are not automatically listed at the new private primary care center. They have to actively choose the new, nearer provider [51]. Västerbotten also follows a similar path as Jämtland in that the county’s financial reimbursement system is predominantly based on capitation (85%) adjusted to age and expected care needs (CNI), while payment per visit (fee-for-service) constitutes a minor part [51]. Overall, there are many similarities between Jämtland’s and Västerbotten’s accreditation rules.

Another similarity is the use of rural allowances, designed as additional reimbursement for listed patients living far away from the nearest hospital [51]. Such extra payments are considered necessary to maintain primary care centers in rural areas. The Social Democratic politician interviewed noted, however, that, since health care provision in rural areas often struggles with a significantly smaller patient base and higher personnel costs, the rural allowance is not enough to compensate health centers in such areas. He stated that it would be possible to increase the sum, but that, in the end, it is hard to provide full financial compensation for what is the basic problem, the low number of inhabitants. The county council in Västerbotten also uses other means to provide better access to care in rural parts of the county. One is to define in-patient care, such as observation and rehabilitation, palliative care, or emergency medical assistance provided by rural health centers as ‘additional services,’ distinct from the regular primary health services included in the accreditation rules [51]. This labeling makes it possible to demand that only primary care centers in rural areas have to provide them. This allows the county to provide extra resources to health centers located in the rural areas and adapt their organization to local conditions (e.g., the long distance to the nearest emergency care hospital) without formally breaking the competition-neutral accreditation rules demanded by the Primary Care Choice Reform.

An additional measure taken by the county to preserve access to care in the rural areas is to, in practice, allow public primary care centers in rural areas to run at a loss, even though this violates the Primary Care Choice Reform’s legal statute of fair competition. The political representative for the majority acknowledges that this is necessary if the rural health centers are to survive:

“By rights, we should let them [the primary care centers in those locations] liquidate. But it is, here in Västerbotten, a political decision to have at least one primary care center in each municipality.” (The political representative for the majority, Västerbotten)

Furthermore, Västerbotten county has informally adopted the policy that if a privately operated primary care center in a rural location is forced to shut down, the county will open a public one in the same location, even if it will run at a loss too. This is to ensure that each municipality has at least one primary care center or hospital.

“If we [the county council] were privately driven, we should go bankrupt on those resorts. But [the county council] Västerbotten has made a political decision that there should be at least one primary care center or one hospital in every municipality.” (Civil servant, Västerbotten)

Taken together, the empirical information gathered through the case study in Västerbotten county shows that the Primary Care Choice Reform was perceived as a challenge in that it is making it more difficult than before for the county to preserve access to care in its rural areas. A particular problem appeared to be the different conditions in the relatively large cities and the remote, scarcely populated areas in the inland parts of the county. Rural allowances appeared to be the most efficient tool for supporting rural primary care centers and thereby fulfilling the political goal of accessible health care for the whole population, even if this measure was believed to be insufficient. What is notable is that the Västerbotten county council, like that of Jämtland, has resorted to measures that in some sense seem to violate the principles of the Primary Care Choice Reform in order to be able to uphold access to care in the most remote areas.

### The case of Västernorrland county

The third case, Västernorrland county, is smaller than Jämtland and Västerbotten, at about 21.7 km^2^ and has a population density of 11 people/km^2^. Like Jämtland, the county has experienced a successive decrease in population since the 1960’s. Just as in Västerbotten, the major cities are located on the coast. Left-wing parties have run the county for most of the post-war period, with the Social Democrats as the leading political actor, but in the election of 2010 the local political scene changed as the left-wing rule was replaced with a center-right coalition. Västernorrland had five private for-profit providers already before the introduction of the reform in 2010. Since then, seven additional private providers have established, all in the coastal cities. Several of the new private primary care centers took over former public ones that were closed down. Altogether, including the public providers, Västernorrland had 32 primary care centers in 2017.^4^

When asked about their perceptions of the Primary Care Choice Reform, respondents in Västernorrland gave a more mixed response than in the other counties. The politician representing the center-right governing coalition was positive towards the reform as he believed that it increases the opportunity for citizens to choose care providers. He did not perceive a clear conflict between the reform and equity in access and did not acknowledge the need for any special measures to protect providers in remote areas from the effects of competition. Instead, he emphasized the county council’s ambition to design the accreditation rules to accomplish fair competition between public and private providers. He was, however, critical of the government’s decision to make the reform compulsory for the county councils. “Because I think… well, it is an infringement of local autonomy.” The politician representing the political opposition, a Social Democrat, questioned the possibility of ‘fair competition’ as the public sector (i.e. the county) always has the ultimate responsibility for primary care provision, including in the rural areas with low population density. According to her, the biggest disadvantage of the reform was that the county council was still financially responsible for all care provision but was now unable to steer the location of providers and allocation of resources to them. She noted that several new private health centers had opened in close proximity in the largest city, but none in the county’s rural areas. Västernorrland had also experienced sudden closures of public health centers as patients had left for new private ones, which had proven costly for the county as it is legally restrained from terminating employment within short notice and may have long-term contracts for the facilities:

“About half of the public deficit of 30 million in 2011 was caused by remaining costs for facilities and personnel (in the closed facilities) …. ...So of course, this jerkiness is costly.” (The political representative for the opposition, Västernorrland)

Both politicians interviewed in Västernorrland noted that there is no real choice for patients in rural parts of the county. Most rural areas have too few inhabitants to support more than one provider. The politician in opposition concluded: “if the private providers … don’t see a market, we don’t argue.” As in the other cases, the politicians interviewed in Västernorrland pointed out that the biggest challenge for providing access to health care in the county’s rural areas is recruiting medical doctors. Västernorrland county has to rely on temporary medical staff hired on a weekly basis for the rural health centers, which is expensive and drains public resources. Often, this means that rurally located health centers can offer fewer GP hours to patients.

#### Measures to promote equity in access to care in Västernorrland

Similar to the cases in Jämtland and Västerbotten, the accreditation rules in Västernorrland state that all primary care centers should provide a relatively broad scope of primary care services, including district nurse care, child- and maternal health care services, psycho-social counseling and home-based health care [52]. In contrast to policy makers in the other cases, however, the political representative of the governing center-right coalition in Västernorrland did not acknowledge the provision of a broad range of services to be a means to support rurally located health centers, but saw it as a way to make available a wide range of services for all patients. Västernorrland’s financial reimbursement system differed from those of Jämtland and Västerbotten in that it had a larger share of reimbursements being based on patient visits, approximately 20% [53]. This reimbursement choice, which favors providers with a relatively large share of visits (typically associated with urban settings) was motivated by the belief that it would increase efficiency and encourage more flexible opening hours, an argument which closely follows the stated goals of the Primary Care Choice Reform [48]. As in the other cases, capitation payments to providers were risk-adjusted in that they provided extra payments for patients above a certain age and with expected higher health care needs according to the care need index [53]. Finally, the financial reimbursement formula in Västernorrland also had, like in Jämtland and Västerbotten, a special allowance for rural locations [53]. The rural allowance, together with the risk adjustments for age, were indicated by the politician representing the center-right governing majority as the main strategies used by the county to support rurally located health centers. As in the other cases, the rural allowance was based on the number of patients living within a certain distance from the closest hospital [53]. On the question of raising the rural allowance, the politician representing the left-wing opposition stated:

“It might, hypothetically, be possible to attract new providers to establish in the rural parts if one raises the rural allowance substantially. But, I don’t know if it is possible to set such a high allowance… because at the same time, that implies, that the urban primary care centers get less money because we cannot add more money into the budget.” (The political representative for the opposition, Västernorrland)

In sum, it appeared that there was somewhat less concern regarding the implications of the Primary Care Choice Reform for equity in access to care in the rural areas in Västernorrland county compared to the other cases. The political leadership expressed a more positive attitude towards the reform, which is not surprising given its political orientation. Even so, it was acknowledged that the reform implied a loss of local autonomy. Regarding measures taken to try to uphold access to care in the rural areas, Västernorrland used similar strategies as in the other cases, such as age-based capitation and rural allowances. A notable difference, however, was that no measures appeared to have been implemented which could be seen to undermine the principle of market competition, for example, in the form of allowing rural health centers to run at a loss or providing extra resources so that they could provide additional services such as inpatient care. Instead, fair and neutral competition rules so as to promote competitive markets appear to have been the overriding policy goal in this case.

## Discussion

The main research question asked at the outset of the paper was how local health authorities in rural Swedish counties, obliged to implement a market-orienting reform, acted to preserve the policy goal of equity in access to care. The three case studies presented in the paper provide a clear illustration of the inherent conflict between a market-based steering logic, embodied through the reform, and the overriding goal of Swedish health care to provide equitable access to primary care services for all citizens, regardless of where they reside. They show that local political representatives and civil servants acknowledged this conflict and the general loss of the capacity of local authorities to steer health service provision as a result of the Primary Care Choice Reform. In order to live up to the reform’s demands for fair competition between all care providers, the county councils had to design accreditation rules and financing formulas which apply to all providers, regardless of their geographical location. This implies that their ability to be flexible and adapt the organization of care provision to shifting conditions, such as between urban and rural areas, was significantly reduced. Counties led by majority left-wing politicians were critical of the reforms, which they viewed as a threat to maintaining access and developing innovative solutions for health care provision in rural areas with low population density. The case studies presented in the article also confirm findings in previous research regarding the general challenges associated with care provision in rural areas, such as a small and shrinking population base, long traveling distances, and the difficulties in recruiting medical staff [2, 25].

A particular aim of the study was to investigate which, if any, measures were taken by local authorities to protect care provision in remote areas while implementing the Primary Care Choice Reform. The case studies identified several such measures, which showed that the county councils did use their autonomy to adjust the contents of the reform to local conditions. These measures can be understood as strategies to protect what was seen as a more fundamental health policy goal, the promotion of equitable access to care for the whole population, including those residing in rural areas. ‘Strategies’ can be understood as planned actions with a distinct long-term goal, in this case to preserve access to care in rural areas by hindering closures of health centers in such areas. The most common strategies used to this end were: formulating the accreditation rules so that a broad scope of services was demanded from all providers seeking to establish in the county; using a relatively high share of capitation in the funding formula so as to not disfavor providers with few patient visits; and a special ‘rural allowance’ to provide extra resources to health centers located far away from the nearest hospital. A broad scope of services implies that inhabitants in rural areas do not have to travel far for health care. Furthermore, by demanding that all health providers offer a broad scope of services, such as maternity care or psychotherapy, the start-up costs for new private providers tend to increase, which may be considered as discouraging. In the two cases with left-wing majorities, there were clear indications of such motives, which also implies that political orientation played a role in how the marketization reform was implemented. In addition to these measures, which were found in all three cases, a few other strategies to protect health centers in rural areas were observed in the case studies, such as allowing them to run at a loss in order to prevent them from closing down; placing some health services offered at health centers in rural areas, such as inpatient care, outside the regulatory framework set by the accreditation rules, thereby making it possible to provide extra funding for them; and, cooperating with other public authorities in rural areas by co-financing certain activities such as district nurses and ambulance care. Such strategies can be seen as violating the principles of the Primary Care Choice Reform in that they offset principles of fair and open competition and equal treatment of all care providers. Taken together, these findings illustrate how reforms initiated at the national policy level can be moderated at the local level, where conditions for meeting sometimes conflicting policy goals can be vastly different from the ‘standard’ conditions alluded to in policy prescriptions. They also provided an illustration of how market reforms, intended to promote values such as competition, economic efficiency, and consumer choice, can be adjusted in various ways to be more compatible with other policy values, such as equity in access or flexibility in service provision.

The case studies presented in the paper also demonstrated that, even though successful measures were taken by local policy makers to uphold access to care in remote areas, the Primary Care Choice Reform was still seen as harmful to the goal of equitable access in that it made it harder to support rural care provision through innovations like cooperation between health centers and municipal social services or the development of e-health. These findings support earlier findings in the literature that geographical equity in health care provision risks being harmed by market-orienting reforms such as privatization of provision or patient choice [14, 17, 19, 20]. It should be noted, however, that there has been no national evaluation of the effects of the Primary Health Care Choice Reform on geographical equity in access in Sweden. What is known is that almost all new (private) establishments of primary care centers after 2010 have been in cities or urban areas. On the other hand, there have been no known closings of health centers in rural areas [17, 20]. This suggests that there has been no change in access in terms of travelling distance to a primary care center for residents in such areas, although they continue to be disadvantaged compared to urban residents.

More generally, the results can also be said to illustrate the, sometimes, conflicting interests between patients living in rural and urban areas. For patients in urban areas, it might be valuable to have new health providers establishing themselves to compete with existing ones, as this may lead to more choice, diversity, and perhaps better services. For patients in rural settings, an increase of providers in the cities tends to drain resources from the countryside, especially if they are both financed through the same public system. As evident from the empirical findings in the paper, such resource shifts can in part be mitigated by higher levels of reimbursement to rurally located providers, but if they are set too high, establishments in the cities, where most patients actually live, are discouraged.

A limitation of the study is that it relies solely on three case studies, which makes it hard to generalize the findings. As all counties in Sweden have different conditions, in terms of geography, demography, and political governance, it cannot be ruled out that different strategies are used in other rural counties. Going beyond the Swedish case, generalization is even more difficult, especially in light of the fact that the Primary Care Choice Reform introduced in 2010 has distinct features that are not likely to be replicated in other countries. What speaks in favor of generalizability, however, is that the experiences from rural health care provision reported in the case studies were highly similar to what has been found in previous research [2, 27]. Moreover, it was striking that the problems experienced with implementing the reform in rural areas were similar in all three cases, as they all were related to the need for flexibility in organization and resource-allocation in order to provide health services in such areas.

## Conclusions

The findings of the article illustrate the challenges involved in introducing market reforms like competition and privatization of rural health care provision, as such policies tend to hinder the ability of public authorities to uphold access to care in such areas. If market-based health care providers choose to locate primarily in the towns, as they did in the Swedish case and have been known to do in other cases, resources will flow in this direction, away from the countryside, and inequalities in access to health services will widen. Market rules honoring principles of fair competition will also risk becoming a strait jacket for local health authorities as they make it difficult to financially compensate rural health providers for their higher costs, which are typically due both to low population density and difficulties in recruiting permanent staff. The findings also show, however, that such negative effects can be moderated. By designing local regulatory systems governing, for instance, service supply, financial reimbursements, and patient listing, market incentives may be altered so that fewer providers choose to locate in the cities, and some even in the countryside. In general, however, the findings of the paper suggest that there is an inherent contradiction between providing health services to patients in rural areas, which typically requires public planning as well as flexibility in use of resources, and creating competitive markets for care provision.

## Footnotes

1 Based on own calculations within the project.

2 Ibid.

3 Ibid.

4 Ibid.
